# Lower Socioeconomic Status and Disability Among US Adults With Chronic Kidney Disease, 1999-2008

**Published:** 2011-12-15

**Authors:** Laura Plantinga, Kirsten L. Johansen, Dean Schillinger, Neil R. Powe

**Affiliations:** Department of Epidemiology, Rollins School of Public Health, Emory University; University of California, San Francisco, California; San Francisco General Hospital, San Francisco, California; San Francisco General Hospital, San Francisco, California

## Abstract

**Introduction:**

Socioeconomic disparities are associated with the prevalence of disability in the general population; however, it is unknown whether a similar association exists between socioeconomic status and disability from chronic kidney disease (CKD, defined as albuminuria or an estimated glomerular filtration rate of 15-59 mL/min/1.73 m^2^).

**Methods:**

A total of 4,257 US adults aged 20 years or older with CKD who participated in the National Health and Nutrition Examination Survey 1999-2008 completed standardized questionnaires assessing self-reported difficulties in activities of daily living (ADL), instrumental ADL (IADL), lower-extremity mobility (LEM), and leisure and social activities (LSA). We used multivariable logistic regression with population-based weighting to obtain adjusted prevalence estimates of disability by demographic, socioeconomic, health care access, and clinical characteristics.

**Results:**

Participants with less education had more disability (age- and sex-adjusted prevalence of disability by lowest vs highest level of education: ADL, 24.5% vs 16.9%; IADL, 34.0% vs 20.3%; LEM, 56.9% vs 44.6%; LSA, 26.2% vs 16.8%; *P* < .001 for all). We observed similar trends for income. After further adjustment for other sociodemographic factors, health care access, and comorbid conditions, education and income both remained significantly associated, by any measure, with lower prevalence of disability.

**Conclusion:**

Among people with CKD in the United States, lower socioeconomic status is associated with greater risk of disability, independent of race/ethnicity, health care access, and comorbid conditions. Our findings suggest that people with CKD and limited education or lower income should be targeted for early intervention to limit disability and further loss of income, both of which could worsen outcomes in CKD.

## Introduction

Disability, as measured by reduced physical functioning (1,2), frailty (3,4), and decreased cognitive functioning (5-8), is commonly reported among people with chronic kidney disease (CKD) and end-stage renal disease. Socioeconomic disparities in both the development and progression of CKD have also been well described (9-12). Socioeconomic factors have been associated with disability in the general population, but this relationship has not been studied among people with CKD (13). Identification of socioeconomic status as an independent risk factor for disability in people with CKD, beyond the high risk conferred by CKD and its risk factors, would provide a means for early identification of those at risk and, possibly, intervention.

Race/ethnicity and other factors that contribute to socioeconomic status (SES), including income and education, could affect both CKD and disability through multiple pathways (Figure). Environmental and personal factors could lead to both CKD and disability; risk factors for CKD and disability, including diabetes and hypertension, could also be heavily influenced by the same factors. Such risk factors could lead directly to disability or could lead to CKD, which in turn could lead to disability through complications of CKD. Additionally, older age is strongly associated with both CKD and disability and could lead to disability directly or through increased presence and severity of comorbid conditions or increased use of medications with age. Finally, disability could affect modifiable sociodemographic factors, particularly income, if the disability interferes with work (14).

**Figure.**
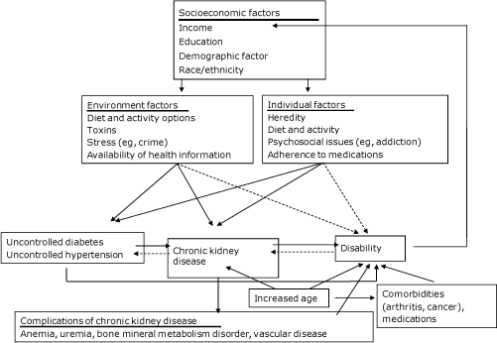
Pathways from socioeconomic and demographic factors to chronic kidney disease and disability. Solid arrows indicate likely paths; dashed arrows indicate possible paths.

Our primary objective was to establish whether a socioeconomic (defined as education level and household income) disparity exists in the prevalence of self-reported disability among noninstitutionalized US adults with CKD, independent of the unique complexity of this disease, which involves many coexisting factors that may contribute to disability. Our secondary objective was to compare these associations with the association of race/ethnicity and disability. We also explored whether age and the severity of CKD modified the association of disability with SES and compared our findings in people with CKD and people without CKD.

## Methods

### Study design

The National Health and Nutrition Examination Survey (NHANES) is an ongoing annual survey conducted by the National Center for Health Statistics (NCHS) of the Centers for Disease Control and Prevention. NHANES consists of a standardized in-home interview and a physical examination at a mobile examination center (MEC). Data from NHANES consist of representative samples of noninstitutionalized, nonmilitary US residents. All survey participants give written informed consent, and the NHANES protocol is approved by the NCHS research ethics review board.

We used 1999-2008 NHANES data. We limited our analysis to 4,305 NHANES participants aged 20 years or older whose serum creatinine, urinary creatinine, and urinary albumin measurements indicated CKD. We excluded participants who did not respond to the physical functioning questionnaire (n = 9), had a low estimated glomerular filtration rate (eGFR) (<15 mL/min/1.73 m^2^) (n = 39), or were pregnant (n = 0 after other exclusions), which gave us a final sample of 4,257.

### Measurements

As part of the home interview, participants are asked via the computer-assisted personal interviewing system about physical function limitations: difficulty performing activities of daily living (ADL) (eg, getting in and out of bed; using a fork, cup or knife; dressing), instrumental ADL (IADL) (eg, managing money, performing house chores, preparing meals), lower-extremity mobility (LEM) (eg, walking a mile, walking up 10 steps, stooping, kneeling or crouching, walking between rooms on the same floor, standing up from an armless chair), and leisure and social activities (LSA) (eg, going to the movies, attending social events, leisure activity at home) (15).

Interviewers obtain self-reported information on demographic characteristics (age, sex, race/ethnicity), socioeconomic status (education, income), health care access (health insurance, routine source of care), health conditions (self-reported diagnosis of diabetes, hypertension, cardiovascular disease [CVD], cancer, and arthritis), and physical activity during the health examinations. Study interviewers record prescription medications from prescription bottles provided by the participant. As part of the MEC examination, health care providers measure height, weight, and systolic and diastolic blood pressure (average of ≥3 auscultatory measurements). Providers also collect serum and urine samples. In accordance with the NHANES study protocol (http://www.cdc.gov/nchs/nhanes/nhanes2007-2008/lab_methods_07_08.htm), laboratory personnel at various sites measure serum creatinine, urinary creatinine, urinary albumin, and hemoglogin and assess C-reactive protein from frozen samples.

### Definitions

We defined disability as a report of "some" or greater difficulty in at least 1 of the activities in the ADL, IADL, LSA, or LEM domains.

We defined CKD as either reduced kidney function or elevated albuminuria. We calculated eGFR according to the modified Modification of Diet in Renal Disease Study equation for calibrated creatinine (16). We defined albuminuria as a urinary albumin-to-creatinine ratio (ACR) of at least 30 mg/g. We defined CKD stages as stages 1 and 2 (eGFR ≥60 mL/min/1.73 m^2^ and ACR ≥30 mg/g on single measurement [evidence of kidney damage]) and stages 3 and 4 (eGFR 15-59 mL/min/1.73 m^2^ on single measurement [evidence of reduced kidney function, with or without kidney damage]).

We defined self-reported diabetes, CVD, cancer, and arthritis by an answer of yes to the question, "Have you ever been told by a doctor or other health professional that you have (disease or condition)?" We further defined self-reported CVD by an answer of yes to the question, "Have you ever been told by a doctor or other health professional that you have coronary artery disease, angina, myocardial infarction, stroke, or congestive heart failure?" We defined hypertension by an answer of yes to the question, "Have you ever been told by a health professional that you have hypertension?" or by measured systolic or diastolic blood pressure of at least 140 or 90 mm Hg, respectively; obesity by a body mass index (calculated from measured height and weight and reported in kg/m^2^) of at least 30; anemia by a hemoglobin of less than 12 g/dl (women) or less than 14 g/dL (men); and inflammation by a C-reactive protein level of at least 1 mg/dL. We categorized physical activity by whether participants self-reported activity levels that they perceived to be higher, the same, or lower than those of their peers.

### Statistical analyses

We summarized selected characteristics and compared excluded participants with those included in the study by these characteristics. We calculated adjusted prevalence estimates and *P* values using multivariable logistic regression models and estimated variance of proportions by using Taylor-series linearization. We chose adjustment variables, including sociodemographic characteristics (age, sex, race/ethnicity, education, income), health care access factors (insurance, routine site for health care), and clinical conditions (diabetes, hypertension, obesity, cardiovascular disease, cancer, and arthritis), based on their demonstrated strong association with disability and sociodemographic characteristics or *a priori* belief that they were confounding factors in the association of disability with CKD. We conducted sensitivity analyses using subgroups of interest, based on age and CKD severity, with further covariates; we explored interaction terms among sociodemographic measures and compared results with those of people without CKD (n = 16,898). We performed all analyses using the survey commands in Stata version 10.0 (StataCorp, LP, College Station, Texas), accounting for study design weights, strata, and primary sampling units. Significance was set at *P* < .05.

## Results

### Characteristics of study population

Among the 4,257 participants with evidence of CKD (85.4%), the mean age was 61 years, and most participants were female, non-Hispanic white, US-born, and married (Table 1). Most (61.0%) had an annual income less than $45,000, although most had an income above the federal poverty level. Most were insured and had a routine site for health care. Comorbid conditions were common, particularly hypertension, arthritis, obesity, cardiovascular disease, and diabetes (Table 1). Participants were evenly divided by CKD severity. Excluded participants (n = 48) were significantly more likely to be nonwhite, to be unmarried, to be nonobese, and to have hypertension, compared with included participants.

### Prevalence of disability

The prevalence of reported disability was high among adults with CKD; 1 in 5 reported limitations in ADL after adjustment for age and sex (Table 2). The highest prevalence was for LEM (50.0%). Higher levels of education and income (measured as annual household income or as a percentage of the federal poverty level) were associated with lower prevalence of disability among all measures examined. Country of birth was associated with prevalence of difficulties with IADL, LEM, and LSA, with lower prevalence among participants born outside the United States. Prevalence of disability was higher in the non-Hispanic black and Mexican American populations compared to non-Hispanic whites only for ADL.

With full adjustment for other sociodemographic, health care access, and clinical variables, prevalence of disability was not associated with race/ethnicity with the exception of ADL disability, which was higher in nonwhite participants (Table 3). Higher education was associated with lower prevalence of reported difficulties with IADL, LEM, and LSA (Table 3). Higher income remained significantly associated with lower prevalence of disability among all measures. Fully adjusted models, including both income and education (*r* = 0.28, *P* < .001), showed that education was significant only for IADL disability: 14.2% for less than high school (reference group for *P* values), 11.8% (*P* = .02) for high school graduate, and 12.2% (*P* = .04) for more than high school.

### Sensitivity analyses

Prevalence of disability was generally higher for older participants (≥65 y) and those with more severe CKD. The association of higher educational attainment with lower prevalence of LEM disability was significant only in participants aged 20 to 64, relative to those aged 65 years or older. Across all measures, the association of higher income with lower disability was strongest in the younger age group (data not shown). Disease severity did not modify the association of educational attainment and higher income with lower prevalence of disability.

There was significantly greater LSA disability among older nonwhites relative to their white counterparts (non-Hispanic blacks, 29.2%, vs non-Hispanic whites, 16.7%, and Mexican Americans, 19.3% vs non-Hispanic whites, 9.0%).

The association gradient of higher education with lower prevalence of ADL disability remained consistent among populations (Table 4). However, people with CKD were, overall, 2 to 3 times as likely as those without CKD to report ADL disability. Regardless of comorbid conditions, the prevalence of ADL disability was higher among those with CKD. Among participants with no other conditions, ADL disability was 2 or more times as high in those with CKD and the lowest educational attainment as in those without.

## Discussion

Disability, among a variety of domains, is prevalent in the US adult population with CKD. Lower educational attainment and lower income were strongly and consistently associated with higher prevalence of disability — that is, among all measures and independent of the other sociodemographic and clinical factors that we measured. Even in the presence of other conditions that can cause disability (eg, diabetes, CVD, cancer, arthritis), comorbid CKD confers a higher likelihood of disability, and this excess prevalence is greatest among people with lower educational attainment. Associations between CKD and SES (9,10,12,17) and between SES and disability (18) have been examined previously. However, to our knowledge, this is the first study to examine SES as a potential risk factor for disability among people with CKD, independent of minority race/ethnicity, older age, and the myriad comorbid conditions associated with prevalence of CKD and disability.

We found that lower educational attainment and lower income, measured as either total income or as income relative to the federal poverty level (http://www.census.gov/hhes/www/poverty/data/threshld/index.html), were inversely related to all measures of disability. This linear association reinforces the presence of a powerful SES health gradient in which health outcomes are worse with lower income, regardless of whether income is above or below the poverty level (13,14,19). Additionally, in older adults, educational attainment was strongly associated with disability, as was lower income, suggesting the association is likely to be in the direction of SES to disability (vs disability to SES), because people with disability may have a subsequent loss in income but not in educational attainment.

For people with CKD, lower SES may lead to disability through poor control of the conditions that cause CKD, particularly hypertension and diabetes, because of the person's lack of understanding of the disease or the resources needed for adequate self-management and treatment. For example, poor blood pressure control, which is common among people with CKD (20,21), could lead to disability not just through stroke but through other systemic effects of hypertension (22). In diabetes-associated CKD, poor glycemic control may also lead to greater risk of disability; one study suggests that retinopathy and neuropathy, sequelae of poor glycemic control, were strong predictors of disability in African Americans with diabetes (23). We found that poor glycemic control did not change the association of education and income with disability in people with CKD and diabetes. Additionally, we showed that people whose CKD was complicated by diabetes and hypertension had higher rates of disability than those with diabetes and hypertension but no CKD, suggesting that CKD has an independent effect on disability prevalence beyond that conferred by these 2 etiologic factors.

Environmental factors, including neighborhood effects (11,24), could also contribute to the observed disparities in disability among people with CKD. Personal psychosocial issues such as depression, which is prevalent among people with CKD (25) and less likely to be successfully treated in people with lower SES (26), may also lead to disability. Lower education level is associated with limited employment opportunities, and available jobs may require intense manual labor or carry increased risk of injury. This situation may partially explain why education affects LEM disability only in the younger (working) population.

Adjustment for comorbidity and medication did not change the effect of education and income on disability, which suggests that these factors are unlikely to explain the associations. Inflammation, anemia, and level of physical activity also did not explain the association. In subgroup analyses, we found that income was more strongly associated with disability in younger (20-64 y) people. This supports the "reverse causality" scenario of decreased physical and mental functioning leading to lower income in people of working age through unemployment, early retirement, or disability. In general, there was little evidence that other sociodemographic factors modified the effect.

In contrast to the associations seen with SES, adjustment for education and income nullified most of the associations of race/ethnicity with disability. The lack of effect of minority race/ethnicity may be due to lack of power in our study; such disparities in disability have been noted in other populations (27,28). However, this lack of effect may also suggest that biological differences that would lead to disparities in disability by race/ethnicity are unlikely and that social status, which is related to race/ethnicity for many, is a more important predictor of disability, at least among people with CKD.

Our study has several limitations. First, because our study was cross-sectional, we cannot establish causality, despite strong indications of causality, including degree of association and consistency of association among measures. Some misclassification of disability and comorbid conditions is possible because these conditions are self-reported. Misclassification of CKD is also possible because of measurement error. The cross-sectional design did not allow assessment of lifetime socioeconomic status, which may be a better predictor of health outcomes than current income (29,30). Also, although CKD is generally a silent disease with few symptoms in its early phases, certain complications of CKD, such as anemia and uremia, could lead directly to disability. We were not able to examine the mechanism by which complications of early-stage CKD lead directly to disability because such complications were rare in our sample. Single assessments of eGFR and albuminuria likely led to misclassification of some participants as having chronic disease; however, any misclassification bias created would be toward the null. Additionally, Mexican Americans were treated as "not African American" in the calculation of eGFR, which may misclassify them, because the equations were developed for primarily white and black non-Hispanic populations.

Despite these limitations, identifying a socioeconomic disparity in disability among people with CKD similar to disparities seen in non-CKD populations may be useful, because both disability and lower SES can worsen CKD outcomes. Because the disparities we identified were independent of age, race/ethnicity, and common comorbid conditions, our findings provide support for a prospective study of the relationship between socioeconomic status and subsequent development of disability among people with CKD. If the associations reported here were confirmed in a longitudinal study with appropriate causal inference, people with lower educational attainment or lower income who are at risk for CKD could be targeted for earlier identification and intervention to limit the development of disability and possible further loss of income due to disability*.* Further exploration of the mechanisms by which lower SES is associated with greater risk of disability among people with CKD is needed to identify effective interventions.

## Figures and Tables

**Table 1:** Characteristics of US Adults^a^ With Chronic Kidney Disease (N = 4,257), National Health and Nutrition Examination Survey, 1999-2008

**Characteristic^a^**	% (95% CI)
**Demographic**	
**Age, y^b^**	
20-39	15.8 (14.2-17.6)
40-59	29.4 (27.4-31.4)
60-69	16.4 (15.1-17.9)
≥70	38.4 (36.2-40.8)
**Sex**	
Male	42.4 (40.8-44.0)
Female	57.6 (56.0-59.2)
**Race/ethnicity^c^**	
Non-Hispanic white	74.4 (71.2-77.4)
Non-Hispanic black	10.2 (8.6-12.1)
Mexican American	5.6 (4.4-7.0)
**Socioeconomic**	
**Education**	
Did not complete high school	28.0 (26.0-30.2)
High school graduate	26.7 (24.7-28.8)
Some college or college degree	45.3 (42.4-48.1)
**Annual household income, $**	
<20,000	27.3 (24.9-30.0)
20,000-44,999	33.7 (31.5-36.0)
45,000-74,999	20.9 (18.7-23.3)
≥75,000	18.1 (15.7-20.7)
**Percentage of federal poverty level^d^**	
<100	14.7 (12.8-16.7)
100-399	57.9 (55.7-60.0)
≥400	27.5 (25.1-30.0)
**Country of birth**	
United States	87.8 (85.6-89.8)
Other	12.2 (10.3-14.4)
**Marital status**	
Not married	45.0 (42.8-47.4)
Married	55.0 (52.7-57.3)
**Health care access**	
**Health insurance**	
No insurance	15.7 (13.6-18.1)
Public insurance	27.8 (25.1-30.6)
Private insurance	56.5 (53.6-59.4)
**Routine site for health care**	
No	6.9 (5.7-8.3)
Yes	93.1 (91.7-94.3)
**Clinical**	
**Chronic kidney disease severity^e^**	
Mild (stage 1 or 2)	47.3 (44.8-49.8)
Moderate to severe (stage 3 or 4)	52.7 (50.2-55.2)
**Smoking**	
Sometimes or never	83.7 (82.1-85.3)
Every day	16.3 (14.7-18.0)
**Obesity**	
Not obese (BMI <30 kg/m^2^)	61.1 (58.8-63.4)
Obese (BMI ≥30 kg/m^2^)	38.9 (36.6-41.2)
**Diabetes**	
No	79.1 (77.1-80.9)
Yes	20.9 (19.1-22.9)
**Hypertension**	
No	29.8 (28.0-31.7)
Yes	70.2 (68.3-72.0)
**Cardiovascular disease**	
No	76.6 (74.6-78.5)
Yes	23.4 (21.5-25.4)
**Cancer**	
No	84.0 (82.6-85.3)
Yes	16.0 (14.7-17.4)
**Arthritis**	
No	57.7 (55.5-60.0)
Yes	42.3 (40.0-44.6)

Abbreviations: CI, confidence interval; BMI, body mass index.

a All factors were self-reported except chronic kidney disease (measured in spot urine [albumin:creatinine ratio] and serum [estimated glomerular function]) and obesity (measured during exam).

b Mean age, 60.8 y (95% CI, 59.9-61.7).

c Participants who listed their race/ethnicity as "other" were included in all analyses but are not presented here because of small sample sizes.

d Federal poverty thresholds for each survey were defined per US Census estimates (http://www.census.gov/hhes/www/poverty/data/threshld/index.html).

e Defined as mild (single albumin:creatinine ratio ³30 mg/g) or moderate to severe (estimated glomerular filtration rate 15-59 mL/min/1.73 m^2^).

**Table 2:** Prevalence^a^ of Disability Among US Adults With Chronic Kidney Disease, by Demographic and Socioeconomic Characteristics, National Health and Nutrition Examination Survey, 1999-2008

Characteristic	% With Disability							

	ADL(95% CI)	*P* Value^b^	IADL(95% CI)	*P* Value^b^	LEM(95% CI)	*P* Value^b^	LSA(95% CI)	*P* Value^b^
Overall	19.9 (18.3-21.5)	NA	25.9 (23.9-28.0)	NA	50.0 (47.6-52.4)	NA	20.6 (18.6-22.8)	NA
**Education**								
Did not complete high school	24.5 (22.1-27.0)	<.001	34.0 (31.1-37.1)	<.001	56.9 (53.3-60.3)	<.001	26.2 (23.4-29.3)	<.001
High school graduate	20.4 (19.0-22.0)		26.6 (24.7-28.6)		50.7 (48.4-53.0)		21.1 (19.2-23.2)	
Some college or college degree	16.9 (15.0-19.0)		20.3 (18.0-22.8)		44.6 (41.7-47.4)		16.8 (14.8-19.0)	
**Annual household income, $**								
<20,000	28.8 (25.0-31.4)	<.001	36.5 (33.0-40.2)	<.001	61.2 (57.9-64.3)	<.001	30.0 (26.8-33.4)	<.001
20-44,999	20.8 (19.2-22.4)		26.9 (24.8-29.1)		51.9 (49.5-54.2)		21.5 (19.5-23.7)	
45-74,999	15.0 (13.3-16.8)		19.1 (17.0-21.4)		42.4 (39.9-45.0)		15.0 (13.1-17.1)	
≥75,000	10.6 (8.5-13.0)		13.1 (10.8-15.9)		33.5 (30.2-37.0)		10.1 (8.2-12.4)	
**Percentage of federal poverty level^c^**								
<100	32.8 (28.4-37.6)	<.001	41.3 (36.4-46.3)	<.001	64.8 (60.7-68.7)	<.001	35.3 (30.7-40.3)	<.001
100-399	20.4 (18.8-22.1)		26.7 (24.7-28.9)		51.2 (48.8-53.7)		21.3 (19.3-23.5)	
400	11.9 (10.1-13.9)		15.9 (13.6-18.6)		37.5 (34.4-40.7)		11.8 (10.0-14.0)	
**Country of birth**								
United States	20.3 (18.7-22.0)	.56	26.6 (24.5-28.8)	.05	50.8 (48.3-53.3)	.01	21.3 (19.2-23.5)	.03
Other	19.1 (15.7-22.9)		21.8 (18.3-25.7)		42.1 (36.5-48.0)		16.7 (13.7-20.3)	
**Race/ethnicity**								
Non-Hispanic white	19.4 (17.5-21.5)	.01	25.6 (23.2-28.2)	.06	50.9 (48.1-53.6)	.93	20.6 (18.3-23.2)	.19
Non-Hispanic black	22.6 (21.1-24.2)		28.4 (26.5-30.3)		51.0 (49.1-52.9)		22.3 (20.6-24.0)	
Mexican American	26.2 (22.4-30.3)		31.3 (27.0-35.9)		51.1 (47.3-54.9)		24.0 (20.5-28.0)	

Abbreviations: CI, confidence interval; ADL, activities of daily living; IADL, instrumental activities of daily living; LEM, lower-extremity mobility; LSA, leisure and social activities.

a Values are adjusted for age and sex. Disability defined as any difficulty reported in ADL (getting in and out of bed, using a fork, cup or knife, dressing oneself), IADL (managing money, house chores, preparing meals), LEM (walking 1 mile, walking up 10 steps, stooping, kneeling or crouching, walking between rooms on the same floor, standing up from an armless chair), or LSA (going to the movies, attending social events, leisure activity at home).

b Indicates differences across categories (without trend), calculated from logistic regression models and marginal post-estimation.

c Federal poverty thresholds for each survey defined per the appropriate US Census estimates (http://www.census.gov/hhes/www/poverty/data/threshld/index.html).

**Table 3:** Fully Adjusted^a^ Prevalence of Disability Among US Adults With Chronic Kidney Disease, 1999-2008, by Education, Income, and Race/Ethnicity

Characteristic	% With Disability							

	ADL(95% CI)	*P* Value^b^	IADL(95% CI)	*P* Value^b^	LEM(95% CI)	*P* Value^b^	LSA(95% CI)	*P* Value^b^
Overall	18.3 (17.2-19.3)	NA	24.5 (23.1-25.9)	NA	44.2 (42.7-45.6)	NA	19.2 (17.8-20.7)	NA
**Education**								
Did not complete high school	19.7 (17.5-21.9)	.06	30.1 (27.6-32.5)	<.001	47.6 (44.6-50.6)	.003	22.5 (20.0-25.0)	<.001
High school graduate	19.1 (16.6-21.6)		23.2 (20.6-25.7)		44.1 (41.4-53.0)		18.9 (16.4-21.4)	
Some college or college degree	16.5 (14.7-18.4)		21.2 (18.8-23.5)		41.9 (39.6-44.1)		16.8 (14.8-18.8)	
**Annual household income, $**								
<20,000	23.1 (20.5-31.4)	<.001	31.3 (28.7-33.8)	<.001	49.8 (46.4-53.1)	<.001	24.8 (21.8-27.8)	<.001
20-44,999	18.2 (15.5-20.9)		24.3 (21.3-27.4)		46.8 (44.1-49.4)		20.1 (17.0-23.2)	
45-74,999	14.3 (11.5-17.1)		19.0 (15.3-22.7)		37.6 (34.0-41.3)		14.4 (11.6-17.2)	
≥75,000	11.6 (8.4-14.8)		14.9 (11.4-18.4)		34.6 (31.2-37.9)		10.1 (7.5-12.8)	
**Race/ethnicity**								
Non-Hispanic white	17.7 (16.4-19.2)	.009	24.2 (22.4-26.0)	.05	51.0 (49.2-52.9)	.84	19.1 (17.3-20.9)	.14
Non-Hispanic black	22.0 (18.8-25.2)		28.1 (24.4-31.7)		46.6 (43.6-49.5)		22.3 (20.6-25.9)	
Mexican American	22.6 (22.4-30.3)		27.8 (23.3-32.3)		44.9 (41.7-48.1)		20.8 (16.9-24.8)	

Abbreviations: CI, confidence interval; ADL, activities of daily living; IADL, instrumental activities of daily living; LEM, lower-extremity mobility; LSA, leisure and social activities.

a Values are adjusted for age, sex, race/ethnicity, routine site for health care, obesity, diabetes, hypertension, cancer, and arthritis. Disability was defined as any difficulty reported in ADL (getting in and out of bed, using a fork, cup or knife, dressing oneself), IADL (managing money, house chores, preparing meals), LEM (walking 1 mile, walking up 10 steps, stooping, kneeling or crouching, walking between rooms on the same floor, standing up from an armless chair), or LSA (going to the movies, attending social events, leisure activity at home).

b Indicates differences across categories (without trend), calculated from logistic regression models and marginal post-estimation.

**Table 4:** Age- and Sex-Adjusted Prevalence of Disability Related to Activities of Daily Living Among US Adults With and Without Chronic Kidney Disease, National Health and Nutrition Examination Survey, 1999-2008

Comorbid Condition	Age- and Sex-Adjusted % With ADL Disability (95% CI)					

	With Chronic Kidney Disease (n = 4,257)			Without Chronic Kidney Disease (n = 16,898)^a^		

	<High School	High School	>High School	<High School	High School	>High School
Overall	24.5 (22.1-27.0)	20.4 (19.0-22.0)	16.9 (15.0-19.0)	11.3 (10.2-12.4)	7.9 (7.3-8.5)	5.4 (4.8-6.0)
Diabetes	35.7 (30.4-41.4)	34.1 (30.6-37.7)	32.4 (26.9-38.5)	23.3 (18.6-28.7)	18.7 (15.7-22.2)	14.9 (11.4-19.2)
Hypertension	26.7 (24.3-29.2)	23.4 (21.9-24.9)	20.3 (18.1-22.8)	17.8 (15.8-20.1)	12.7 (11.7-13.9)	8.9 (7.8-10.2)
Cardiovascular disease	37.7 (33.0-42.7)	33.7 (30.8-36.8)	30.0 (25.3-35.0)	33.5 (28.3-39.0)	26.3 (23.3-29.5)	20.1 (16.4-24.5)
Cancer	30.3 (22.7-39.3)	26.1 (21.4-31.4)	22.3 (17.9-27.4)	22.0 (17.2-27.7)	16.0 (13.6-18.7)	11.4 (9.2-14.0)
Arthritis	36.9 (33.1-40.8)	33.0 (30.7-35.4)	29.4 (26.0-33.1)	30.6 (27.5-33.9)	23.5 (21.8-25.4)	17.7 (15.5-20.1)
None^b^	10.0 (4.6-20.5)	4.6 (2.6-7.9)	2.0 (1.0-4.2)	3.6 (2.8-4.7)	2.4 (2.0-2.9)	1.6 (1.2-2.1

Abbreviations: ADL, activities of daily living; CI, confidence interval.

a None of the conditions listed above.

b All changes were significant at *P* < .001 (calculated by using general linear model).
