# Erratum, Vol. 9, Dec. 15 Release

**Published:** 2012-01-26

In the article, “Lower Socioeconomic Status and Disability Among US Adults With Chronic Kidney Disease, 1999-2008,” two errors occurred:

The suggested citation for the article should read as follows:

*Suggested citation for this article*: Plantinga LC, Johansen KL, Schillinger D, Powe NR. Lower socioeconomic status and disability among US adults with chronic kidney disease, 1999-2008. Prev Chronic Dis 2012;9:110052. DOI: http://dx.doi.org/10.5888/pcd9.110052

In reference 2, the article title was omitted. Reference 2 should read as follows: 

2. Curtin RB, Lowrie EG, DeOreo PB. Self-reported functional status: an important predictor of health outcomes among end-stage renal disease patients. Adv Ren Replace Ther. 1999;6(2):133-140. 

These corrections were made to our website on January 26, 2012, and appear online at http://www.cdc.gov/pcd/issues/2012/11_0052.htm. We regret any confusion or inconvenience this error may have caused.

